# Conference Report: NIST SPECIATION WORKSHOP Gaithersburg, MD June 13–15, 1995

**DOI:** 10.6028/jres.101.069

**Published:** 1996

**Authors:** M. K. Schultz, W. C. Burnett, K. G. W. Inn, J. W. L. Thomas, Zhichao Lin

**Affiliations:** Environmental Radioactivity Measurement Facility, Department of Oceanography, Florida State University, Tallahassee, FL 32306-3048; Radioactivity Group, Ionizing Radiation Division, Physics Laboratory, National Institute of Standards and Technology, Gaithersburg, MD 20899-0001

## 1. Introduction

The focus of the NIST Speciation Workshop, which was held at NIST on June 13–15, 1995, was the partitioning of radioactive elements in NIST natural matrix standards.

### 1.1 Significance of Soil Fractionation

The National Institute of Standards and Technology radionuclide Standard Reference Material (SRM) program has been successful in making available to the community natural matrix materials that are very useful for the evaluation of radiochemical-measurement techniques. Traditionally, measurements of environmental radiological contamination have focused on the determination of total concentrations, a very useful tool for initial site characterization of impacted areas. It is clear, however, that total concentration does not describe the environmental behavior or bioavailability of contaminating radionuclides. Rather, the time-dependent spread of radiological contaminants is a function of “partitioning” or “speciation” of radionuclides within soils and sediments.

In the United States and many other parts of the world, there remains an enormous task ahead for remediating radiologically contaminated environments and monitoring the impact of man-made radioactivity on the natural environment. The U.S. Department of Energy (DOE) faces contamination plumes of greater than 2.3×10^6^ m^3^ of contaminated groundwater and greater than 150×10^6^ m^3^ of contaminated soil (Wyrick, SAIC, workshop presenter). Furthermore, current technologies for cleaning up and preventing further migration of radioactive contaminants are costly and often ineffective. The development of strategies for remediation, restoration, and mitigation of radiologically contaminated areas is necessarily constrained by budgetary concerns. Therefore, for long-term risk assessment analyses, regulatory bodies need information which takes the mobility and bioavailability of radiological contaminants into consideration. Although the need exists for the continued development of more accurate, efficient, and sensitive radioanalytical techniques, there is also a need for more information concerning what fraction of the contamination in a given environmental sample is “environmentally available.” Presently, there is no acceptable measure of the bioavailability of radioactive elements in contaminated soils and sediments [1].

### 1.2 The Sequential Extraction Approach

The concept of macro- and micro-nutrient metal fractionation or partitioning in soils and sediments has a long history. Soil science as a discipline began to emerge in the late seventeenth and early eighteenth centuries when studies were primarily concerned with soil productivity for agricultural purposes. One of the important findings of the time was the discovery by German physical chemist Julius von Liebeg that crop yields were directly related to the mineral content of a soil [2]. Although this finding essentially revolutionized soil science, it was not the complete story. The flaw in Liebeg’s theory, as discovered by J. H. Gilbert, J. B. Lawes, and others, was that it was not only the total mineral content which governed the productivity of a soil but rather the chemical form of the nutrient within the soil that determined the “availability” of the nutrient to plants [2]. This realization led to the modern concept of “fractionation” of soils. Just as the chemical form of nutrients determines their bioavailability to plants, the mobility and/or bioavailability of radioactive elements is determined by their geochemical associations with the various components of a soil or sediment. The concept of fractionation can thus be applied to the study of the environmental behavior of radioactive elements in soils and sediments.

The primary concerns associated with radionuclides in the environment are (1) migration through natural systems; and (2) bioavailability to organisms *via* the food chain. The concern is that ionizing radiation in sufficient doses can adversely affect a variety of biological processes in higher organisms. The mobility and/or bioavailability of radioactive elements in the environment is dependent upon the element’s physico-chemical form, referred to here as the element’s speciation. These physico-chemical forms, which include the geochemical associations of radionuclides in soils, sediments, and groundwaters, are key factors in understanding the migrational behavior and bioavailability of trace metals and radionuclides [3–11]. These characteristics are dependent upon both the waste medium into which radioactive contaminants are incorporated and the changing *in situ* conditions of the discharge area (soil pH, temperature, mineral composition, etc.).

One approach to identifying the speciation of a given radionuclide within a specific soil or sediment is by sequential extraction. This technique evaluates the leachability of the radionuclide by the application of operationally defined chemical treatments to analyze selectively specific classes of the geological components of soils and sediments. The results then can be used to determine which contaminated sites are of greatest concern. The most cost-effective mitigation and remediation strategies can thus be derived because the speciation and bioavailability of radionuclides have been taken into consideration. Considering the large number of radiologically contaminated sites, the development of such a prioritized approach is highly desirable.

## 2. Workshop Summary

The workshop was divided into three parts: (1) scope of the workshop and benefits to users of NIST SRMs; (2) definition of the “state of the art” in the use of sequential leaching techniques; and (3) development of an experimental plan for a standard leach method of NIST soil and sediment SRMs. Presentations of both published research and “work in progress” were made in reference to each part of the workshop (Table 1). For development of a work plan, participants formed three working groups for deliberations which ultimately led to consensus for an experimental design to optimize the proposed leaching protocol.

## 3. Workshop Goals, Potential Benefits, and SRM User Applications

An introduction to the scope and goals of the workshop was provided by Kenneth G. W. Inn of the Radioactivity Group at NIST. Inn acknowledged that the demands of SRM users have become more sophisticated over the years. In keeping with the needs of SRM users, the NIST Radioactivity Group has begun to explore the possibility of certifying SRM soils and sediments for specific fractions as well as for total concentration of an analyte. NIST “s initial approach to identifying the physicozchemical?associations of a given radionuclide within a specific soil or?sediment is by the application of the chemical sequential?extraction or “fractionation” approach. Thus, the main goals of the workshop were (1) to develop a consensus set of operationally defined fractions that can be selectively separated by chemical means before analysis; and (2) to evaluate the experimental variables (reaction period, temperature, reagent concentration, etc.) so as to optimize the extraction protocol. The hope here is that if laboratories apply a simple standardized procedure to a well-characterized SRM, one can make meaningful interlaboratory comparisons. The scope of the workshop discussions was limited to soils and sediments which are contaminated with either actinide, fission, or activation product nuclides.

**Fig. 1:**
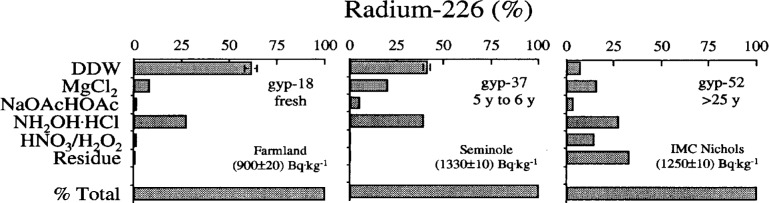
Extraction profile for radium in three phosphogypsum samples of different age showing how speciation is affected by weathering. The “% total” refers to the sum of the ^226^Ra determined in the individual fractions (by ^222^Rn emanation) compared to an analysis of ^226^Ra in the bulk sample (by alpha spectroscopy) [12]. Errors shown are 1 σ based on counting statistics. *y*-axis titles refer to: DDW (double distilled water); MgCl_2_ (magnesium chloride); NaOAc/HOAc (sodium acetate in acetic acid); NH_2_OH · HCl (hydroxylamine hydrochloride); HNO_3_/H_2_O_2_ (nitric acid/hydrogen peroxide solution); and Residue (total dissolution by NaOH fusion) (Burnett et al., 1996). The symbol “y” denotes years.

The potential benefits and operational limitations of sequential chemical extraction techniques were introduced by William Burnett of the Department of Oceanography at Florida State University. The potential benefits to SRM users are both environmental and economic. Risk assessment, mitigation strategies, and the execution of effective long-term monitoring may critically depend on information relating to speciation of radionuclides within contaminated soils and sediments. Billions of dollars in remediation costs could be saved and social concerns addressed as a result of reliable and interpretable radionuclide speciation data. As an example, the mobility of ^226^Ra in phosphogypsum stacks in central Florida is a matter of considerable concern to residents of the region. Phosphogypsum (CaSO_4_ · 2H_2_O), a radium-rich by-product of phosphate fertilizer production, is produced at a rate of approximately 30 million tons per year, which is nearly all stored because of radioactive impurities [12]. Recently, plans have been made by the State of Florida to cover these stacks to prevent leaching of radium into the underlying aquifers. Results of recent research show that the leachability of radium may be reduced over time implying that in some cases this remediation may be unnecessary (Fig. 1).

**Fig. 2:**
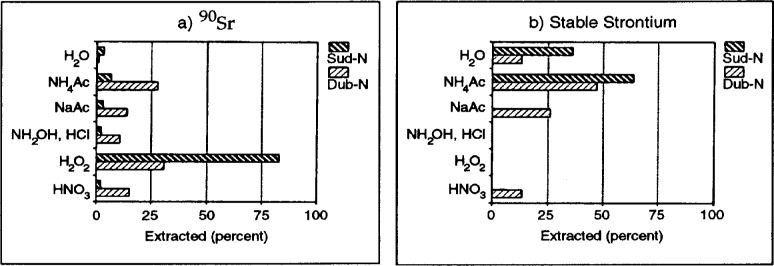
Extraction profiles for radioactive and stable isotopes of Sr in soils from Belarus, Russia [9].

Work on Chernobyl soils presented by Brit Salbu of the Agricultural University of Norway clearly illustrates the unique information which may be obtained by sequential extraction techniques. Her studies have shown another example where the mobility and/or bioavailability of contaminating radioactive elements in the environment may be time dependent. This type of effect may be very important to decision-making with regard to monitoring and/or remediation. Fractionation experiments conducted on soils surrounding the Chernobyl (Ukraine) and Sellafield (United Kingdom) reactor sites indicate that primary associations and behavior of radionuclides associated with fuel particle release may change over time [7–9]. Research conducted by Salbu and her colleagues indicates that the mobility of ^90^Sr (normally found in a highly-mobile cationic species) was initially quite low because of its association with fuel pellets (Fig. 2). However, as the refractory particles began to break down during weathering, the mobilization of ^90^Sr appeared to increase as radioactive Sr moved toward chemical equilibrium with stable strontium in the environment.

**Fig. 3:**
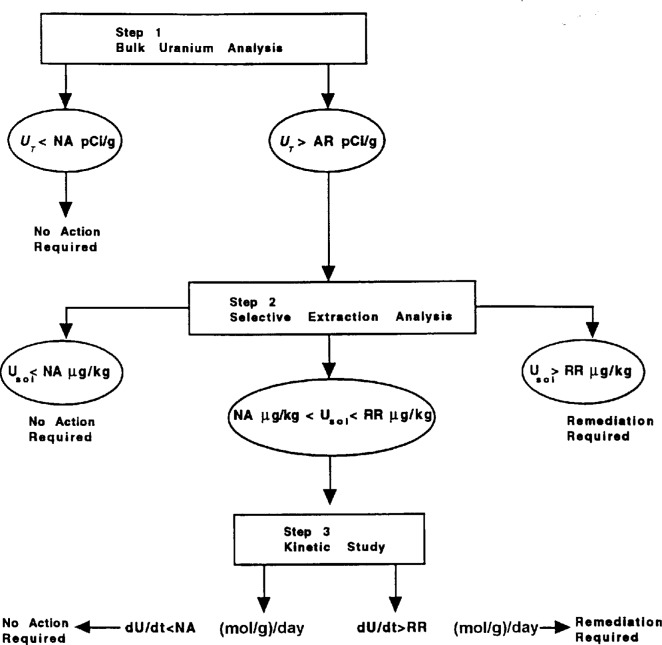
A decision tree for assessment of environmental availability of uranium in soils and sediments [1]. Abbreviations used in this table refer to: NA (No Action Required); AR (Action Required); and RR (Remediation Required) (adapted from Amonnette et al., 1994).

Several presentations addressed the issue of environmental availability with regard to risk assessment and effective remediation management. Environmental availability can be defined as the ability of a soil or sediment to supply (or release) contaminants to points of human contact or to the surrounding environmental media. This involves all processes by which soil (or sediment) contaminants become available for uptake by organisms. The general consensus of the group was that current strategies for remediation and monitoring were both ineffective and costly. Speakers from the several federal agencies and national laboratories each identified the need for more sophisticated information regarding speciation of radiological contaminants as critical to the development of more cost-effective, environmentally sound remediation strategies. For example, James Amonnette of Pacific Northwest Laboratories suggested the development of a decision tree which could be used to assess the environmental availability of uranium in a specific geographical location (Fig. 3). In this approach, the results of selective extraction analysis is combined with bulk sample analysis and kinetic experimental results to determine if remediative action is required. In this way, a hierarchy of geographical areas which require remediative action can be determined and systematically prioritized.

## 4. Definition of the State of the Art

The present situation concerning the use of sequential extraction techniques and the type of analytical results obtainable by their application were highlighted in presentations by several workshop participants of completed and ongoing research. Many of the techniques are derived from earlier works, such as those of Jackson [13], Gupta and Chen [14], and Tessier et al. [15]. A total of 12 modified sequential extraction protocols were presented, which accounted for variations in geochemical composition from sample to sample. Although sample-specific dependencies are a concern, the group felt that a carefully developed analytical protocol could be formulated that would accommodate these variations.

Sequential extraction methods have been used extensively and for decades, as tools for determining the distribution of nutrient elements, transition metals, radionuclides, and other components within various sample matrices. In principle, these techniques can provide insights into the biogeochemical and/or diagenetic processes which affect trace metal and/or radioelement fractionation. However, there are potential problems (i.e., bias introduced by the operationally defined leaching procedures) which must be recognized and avoided if at all possible. The interpretation of results obtained from the application of sequential extraction techniques has often evoked considerable controversy because of these potential pitfalls. From a geochemical viewpoint, sequential extraction procedures have been criticized for both nonselectivity of the target phase(s) [11, 16–19] as well as unquantified read-sorption (sometimes referred to as redistribution) of analyte during the reaction period [11, 16–20]. Although proponents of sequential extractions methods maintain that each reagent phase attacks a specific geochemical fraction of the sample, it is unlikely that certain minerals would be uniquely attacked to exclusion of all others by the reagent. Readsorption or redistribution occurs when an analyte is released during an extraction step but is redistributed or adsorbed onto the remaining mineral phases during the procedure, thereby producing ambiguous results. In addition, several discussions supported the notion that “extractability” is not necessarily an indicator of “bioavailability.” Experimental results from sequential-extraction experiments conducted on Chernobyl soils by Brit Salbu and colleagues, and on lake sediments from a former cooling reservoir at the Savannah River Site by Thomas Hinton, Savannah River Ecology Laboratory, were presented at the workshop. Their results suggest that the “exchangeable fraction” should not be over interpreted to mean “plant uptake” (i.e., biota may exhibit selective ingestion of dissolved contaminants) and caution should be exercised when equating physico-chemical or biological processes to operationally defined fractions. These problems have, in fact, been acknowledged by many supporters of the approach [17], including Salbu and Hinton, who recognize the operationally defined limitations of the sequential extraction approach. In particular, they point out that the partitioning of metals is inherently influenced by such factors as the reagent of choice, time of extraction and ratio of extractant to sample.

The variability of results on the same samples obtained by differing sequential leaching methods was addressed by Susan Clark of the Savannah River Ecology Laboratory. The results of Clark’s research showed that when two different sequential extraction methods were applied to the same contaminated sample from the Savannah River Site, the amount of “available” contaminants varied by as much as 30 %. This type of variability emphasizes the need for a standard approach as well as a rigorous evaluation of the experimental parameters involved.

Another important point to consider in the development of an acceptable extraction protocol relates to the fractional resolution of the method or, in other words, the number of operationally defined fractions which are attempted. While some sequential extraction procedures consist of up to 10 geochemically attributed fractions [14], extraction procedures employing as few as three fractions have provided very useful experimental results. For example, in an investigation of trace metal cycling in the Sargasso Sea, Landing and Lewis [10] used a simple three-step sequential extraction procedure (“easily soluble” acetic acid, “less labile” dilute aqua regia, “refractory” concentrated HCl, HNO_3_, HF) to study geochemical cycling of Fe, Mn, and Al in the water column. Their water column data in the study area showed that particulate Al was dominated by a refractory fraction except at two depths where a more soluble fraction indicated geochemical cycling of Al. In contrast, the Mn profile was dominated by a broad maximum at mid-depth of the more easily leached fraction, with the more refractory fractions in the surface and bottom waters. In addition, zones of geochemical cycling for Fe in the water column were identified by the ratio of leachable to more refractory Fe in particulates. These results, presented at the workshop by William Landing from Florida State University, suggest that the application of sequential extraction methods which utilize a minimum number of fractions, may be less prone to problems while still providing valuable speciation information.

## 5. Recommended Protocol

A major goal of the workshop was to derive a recommended protocol for the sequential extraction of NIST SRM soils and sediments. Since the partitioning of radionuclides by sequential extraction procedures will result in “operationally defined” fractions, the group felt that development of a standard methodology is crucial. In order to achieve this goal, the participants were divided into three working groups (Table 2) and each group was asked how they would improve the well-known Tessier et al. [15] method.

The results of these working group deliberations were then debated in an open forum and a consensus was reached (Table 3). All participants agreed that an empirically based sequential extraction approach would be most likely to result in meaningful information at a reasonable cost. Reagents were chosen which would effectively separate the most important geochemical features of soils and sediments within operationally defined limits of interpretation. When selecting the number of fractions, participants considered the operationally defined nature of the method on the one hand, while still recognizing the need to achieve an acceptable degree of resolution on the other. A deionized water-soluble fraction (usually used as the first fraction) was considered but rejected in favor of a somewhat more compact procedural design. Instead, an overnight sample soak period (wetting of the sample with deionized water), following dry weight determination, was added at the beginning of the extraction sequence to allow for swelling of clay minerals and to bring the sample into a more “natural” state. In addition, it was agreed that the order of extraction in a sequential procedure may play an important role in the “selectivity” of the method and in correct interpretation of results. Solid particles in soils and sediments, for example, often are coated with a layer of organic matter. Organic matter is recognized as an important sink for contaminating radionuclides in the environment, and the possible relationship of radionuclides with organic matter in a sample is thus of vital importance. Furthermore, it is also possible that organic coatings may inhibit the reaction of the other geochemical phases which are targeted by a subsequent reagent in the experimental sequence. The participants agreed, therefore, that the extraction of the “organic” fraction should be placed immediately after the extraction of the “exchangeable” fraction. The reagent recommended for this modified organic fraction was also changed from HNO_3_/H_2_O_2_ as recommended by Tessier et al. [15] to NaOCl. Experimental data have shown that this reagent should dissolve organic matter more effectively and with minimal crossover to other geochemical phases [21]. It was further agreed that separation of aqueous and solid phases for all extractions will be by high-speed (>10 000 *g* if possible) centrifugation followed by filtration of the supernatant solution using a 0.1 mm filter.

The group felt that a defensible sequential extraction method would require that “optimum conditions” for each sequential fraction be experimentally determined. Four experimental parameters (reagent concentration, reagent to sample ratio, duration of extraction period, and temperature of reaction) were identified as potentially significant in terms of obtaining these “optimum conditions.” An experimental plan was designed to test three of these four parameters for each extraction with three possible settings (Table 3). “Optimum conditions,” in this case, refer to the conditions under which problems associated with sequential extraction methods (readsorption, incomplete dissolution, nonselectivity) are minimized or eliminated. Readsorption can be evaluated by the application of “double spiking” isotopic techniques. As an example, consider the quantification of uranium readsorption during the “exchangeable” extraction. Along with the extraction reagent (MgCl_2_), an isotopic tracer (^236^U for example) is added to the sample prior to the reaction period. Upon completion of the reaction period, solid and aqueous phases are separated by centrifugation and filtration, and a second isotopic tracer (^232^U for example) is added to the aqueous phase extractant solution prior to elemental chemical separations. Since the alpha decay energies of ^236^U and ^232^U can be easily resolved by alpha spectrometry, the chemical recovery of ^232^U can be used to quantify the adsorption of ^236^U onto solid-phase particles during the extraction procedure. The ^236^U tracer acts as an indicator of natural uranium readsorption behavior during the procedure. Another very important tool for assessing the completeness and specificity phase of separations during sequential extractions, is analysis of stable elements (Ca, Mg, Fe, Mn, Al, Zr, Sr, and Cs) which can be used as indicators of which phases are being attacked during the reaction.

## 6. Experimental Work

The initial experimental evaluation of the recommended sequential extraction protocol and the determination of the optimum experimental conditions will be carried out by researchers at NIST and Florida State University in a joint project. The sample chosen for the initial development is NIST SRM 4350B, Ocean Sediment, a composite of Irish Sea and Chesapeake Bay sediments, that is being certified by NIST for U, Pu, and Sr. A round-robin intercomparison study by participating workshop laboratories will follow this protocol development to determine the robustness and interlaboratory reproducibility of the method. And finally, the resulting method will be used to provide speciation characterization of future NIST natural matrix radionuclide standards.

## Figures and Tables

**Table 1:** Research topics presented at athe NIST Speciation workshop June 13–15, 1995

Author	Affiliation	Title
Rateb Abu-Eid	NRC	Environmental Availability and Speciation of Uranium in Soils and Sediments, and the Influence on Risk Assessment
James Ammonette	Pacific Northwest Laboratories	Speciation of Uranium in Soils
John Griggs	EPA	Compatibility of Analytical Screening Methods
Steven Wyrick	DOE/EM/SAIC	Speciation Knowledge of Cost Effective Remediation—DOE’s Perspective
Brit Salbu	Agricultural University of Norway	Research on Chernobyl Soils
Lawrence Shuman	University of Georgia	Speciation of Metals in Soils
William Landing	Florida State University	Speciation of Metals in Marine Sediments
Joylene Thomas	NIST	^90^Sr Speciation in NIST River Sediment and Rocky Flats Soil-1, SRM 4350B and 4353
Michael Schultz	Florida State University	Actinide Speciation in IAEA-135 Irish Sea Sediment
Susan Clark	Savannah River Site Ecology Laboratory (University of Georgia)	Extraction Techniques for Savannah River Site Soils
Kurt Bunzl	GSF-Institut fζr Strahlenschutz Neuherburg, Germany	Association of Chernobyl-Derived ^239/240^Pu, ^241^Am, ^90^Sr, and ^137^Cs with Organic Matter in the Soil and Soil Solution
Robert Fjeld	Clemson University	Radionuclide Sorption in Idaho National Engineering Laboratory Interbed Soils and Basalts
Thomas Hinton	Savannah River Site Ecology Laboratory (University of Georgia)	Uptake of Ca by Plants Compared to Operationally-Defined Availability from Sequential Extraction Results
Jordi Vives Batlle	University of Pittsburgh	Characterization of Radionuclide Species in the Environment

**Table 2:** Composition of the three working groups at the NIST Workshop. Each group was asked to evaluate and improve a common strawman protocol

Group 1	Group 2	Group 3
Chet Francis (Chair)	James Field (Chair)	David Piper (Chair)
Joylene Thomas (Recorder)	William Burnett (Recorder)	Michael Schultz (Recorder)
Zhichao Lin (Recorder)	Susan Clark	Kurt Bunzl
James Amonnette	Rateb Abu Eid	Joan Connolly
Dennis Kelsh	Meredith Newman	Isabel Fisenne
Dirk Gombert	Thomas Hinton	J.M. Robin Hutchinson
Kenneth Inn	John Leyba	William Landing
Sy Lee	Brit Salbu	Steven Wyrick
Steven Serkiz	Richard Wells	Jordi Vives Batlle
Nancy Trahey		Lawrence Shuman

**Table 3:** The consensus method for determination of the speciation of actinide elements in soils and sediments. The optimum conditions (reagent concentrations, duration of extraction period, and temperature of reaction) are to be determined by systematically varying the experimental conditions

Fraction	Reagent	Reagent/Sample Ratio	Reagent Conc. (mol/L)	Temp. (°C)	Time (h)
H_2_O/Exchangeable	MgCl_2_	5:1 / 9:1 / 15:1	0.1 / 0.4 / 1.0	25	1 / 2 / 4
Organics	NaOCl, pH 8.5	5:1 / 9:1 / 15:1	5 %	25 / 50 / 95	0.5 / 1 / 2
Carbonates	NaOAc in 25 % HOAc, pH 5	5:1 / 9:1 / 15:1	1	25 / 50 / 95	2 / 4 / 6
Oxides	NH_2_OH · HCl in HNO_3_, pH 2	15:1	0.01 / 0.04 / 0.1	25 / 50 / 95	1 / 4 / 16
Acid/Sulfide	HNO_3_	5:1 / 9:1 / 15:1	4 / 8 / 16	95	1 / 4 / 16
Residue	HF–HClO_4_–HCl or NaOH fusion	n/a 5:1	n/a n/a	Fumed 500	a 3

a The dissolution time is sample dependent.

General Notes:

Each fraction is extracted two times, followed by a rinse with extractant and then two deionized water rinses which are combined with the extractant.

Each reagent-solid mixture will be separated by high-speed (>10,000 *g*) centrifugation followed by 0.1 μm filtration.
